# Chlorophyll to zeaxanthin energy transfer in nonphotochemical quenching: An exciton annihilation-free transient absorption study

**DOI:** 10.1073/pnas.2411620121

**Published:** 2024-10-08

**Authors:** Tsung-Yen Lee, Lam Lam, Dhruv Patel-Tupper, Partha Pratim Roy, Sophia A. Ma, Henry E. Lam, Aviva Lucas-DeMott, Nicholas G. Karavolias, Masakazu Iwai, Krishna K. Niyogi, Graham R. Fleming

**Affiliations:** ^a^Department of Chemistry, University of California, Berkeley, CA 94720; ^b^Molecular Biophysics and Integrated Bioimaging Division, Lawrence Berkeley National Laboratory, Berkeley, CA 94720; ^c^Graduate Group in Biophysics, University of California, Berkeley, CA 94720; ^d^Department of Plant and Microbial Biology, University of California, Berkeley, CA 94720; ^e^HHMI, University of California, Berkeley, CA 94720; ^f^Innovative Genomics Institute, University of California, Berkeley, CA 94720; ^g^Kavli Energy Nanoscience Institute, Berkeley, CA 94720

**Keywords:** photosynthesis, photoprotection, exciton annihilation

## Abstract

Zeaxanthin (Zea) is a key component in the energy-dependent, rapidly reversible, nonphotochemical quenching process (qE) that regulates photosynthetic light harvesting. Previous transient absorption (TA) studies suggested that Zea can participate in direct quenching via chlorophyll (Chl) to Zea energy transfer. However, the contamination of intrinsic exciton–exciton annihilation (EEA) makes the assignment of TA signal ambiguous. In this study, we present EEA-free TA data using *Nicotiana benthamiana* thylakoid membranes, including the wild type and three NPQ mutants (*npq1*, *npq4*, and *lut2*) generated by CRISPR/Cas9 mutagenesis. The results show a strong correlation between excitation energy transfer from excited Chl Q_y_ to Zea S_1_ and the xanthophyll cycle during qE activation. Notably, a Lut S_1_ signal is absent in the *npq1* thylakoids which lack zeaxanthin. Additionally, the fifth-order response analysis shows a reduction in the exciton diffusion length (L_D_) from 62 ± 6 nm to 43 ± 3 nm under high light illumination, consistent with the reduced range of exciton motion being a key aspect of plants’ response to excess light.

## Significance

Nonphotochemical quenching (NPQ) mitigates photooxidative damage inherent to oxygenic photosynthesis. However, the biophysical mechanism of this photoprotection remains contentious, despite longstanding knowledge that zeaxanthin and lutein are somehow involved in quenching excited chlorophyll. Using gene-edited *Nicotiana benthamiana* NPQ mutants and annihilation-free transient absorption spectroscopy, we directly observed a role for zeaxanthin but not lutein in excitation energy transfer and subsequent quenching of excited chlorophyll. By isolating higher-order nonlinear signals, we also validated a relationship between NPQ and exciton diffusion length, bridging our fundamental understanding of land plant NPQ with exciton dynamics in thylakoid membranes.

Safe dissipation of excess absorbed sunlight is essential to the survival and productivity of oxygenic photosynthetic organisms (1–4). The overall dissipative process is known as nonphotochemical quenching (NPQ), which involves the de-excitation of singlet excited chlorophyll (^1^Chl*) via a number of mechanisms with differing timescales of response (5). The most rapid response, which takes place on a timescale of a few seconds to minutes, is termed energy-dependent quenching (qE) and is usually the largest component of the NPQ response. qE depends on the presence of a pH-sensing protein called PsbS in vascular plants (6) and on an enzymatically driven cycle in which three xanthophylls, violaxanthin (Vio), antheraxanthin (Ant), and zeaxanthin (Zea), are interconverted depending on the light conditions. In excess light, the enzyme violaxanthin de-epoxidase (VDE) converts Vio via Ant to Zea. In low light, the enzyme zeaxanthin epoxidase (ZEP) carries out the reverse reactions; Zea→Ant→Vio. The whole system is known as the VAZ cycle (7, 8).

Despite the demonstrated importance of an optimized photoprotective response to sustain and improve crop yields (4), the underlying molecular mechanisms of qE and NPQ in general remain controversial (9, 10). For example, the importance of lutein (Lut) and Zea and their specific modes of action are extensively debated (11). Lut has been suggested to quench ^1^Chl* by an excitation energy transfer (EET) (9, 12) or charge transfer mechanism (13, 14). Lut has also been shown to quench Chl in its triplet state (^3^Chl*), a potent photosensitizer formed from ^1^Chl* via intersystem crossing (2, 15, 16). Zea, on the other hand, has been observed to form both a radical cation (via charge transfer with Chl) and an excited singlet state (via energy transfer from the Chl Q_y_ state) in the heterokont alga *Nannochloropsis oceanica*, although this organism lacks PsbS and requires LHCX1 for qE instead (17, 18). *N. oceanica* does not contain Lut, which enabled a quantitative model to be built based on the VAZ cycle alone (19, 20). The model proposed that the pH sensor is responsible for activation of pigment–protein complexes to active quenching forms. Zea has also been suggested to play an allosteric role, rather than an explicit quencher role, by aiding the aggregation of LHCII complexes, which have shortened fluorescence lifetimes (21).

The role of carotenoids (Car) in NPQ via Chl→Car EET can be explored by tracking the population of the Car S_1_ state after Chl excitation using transient absorption (TA) spectroscopy (9, 17, 18, 22–26). However, because the Q_y_ (S_1_) to S_n_ absorption spectrum of Chl covers most of the visible spectrum, detecting additional contributions of, for example, Car S_1_ to S_n_ transitions demand good signal to noise level TA spectra, particularly for a highly scattering sample such as the thylakoid membrane. This, in turn, requires excitation pulse energies that are significantly higher than those used for fluorescence lifetime measurements. With the required excitation energies, exciton–exciton annihilation (EEA) is almost unavoidable in extended exciton transport systems such as the thylakoid membrane (10, 27, 28), raising concern that any additional transient species observed in TA measurements may simply be the result of high-energy species formed during the annihilation process (29). Recently, however, Maly et al. have demonstrated a remarkably straightforward way of isolating the third order (single particle), fifth order (two particle), seventh order (three particle), etc., contributions to the nonlinear pump–probe signal (30–32). This method enables the extraction of the excited state dynamics free from the multiparticle kinetics such as EEA and increases our confidence in assigning the transients observed during the response of thylakoid membranes to high light. In addition, the 5th-order contribution to the TA signal contains information about the exciton motion, which we analyze briefly in this work.

A second aspect of our earlier work on spinach thylakoids (23) where NPQ mutants are not available is also addressed in this study through the generation of *Nicotiana benthamiana* NPQ mutants. In combination, the TA data and snapshot fluorescence lifetime data collected on key mutants [*npq4* lacking PsbS (6), *npq1* lacking zeaxanthin (33), and *lut2* lacking lutein (34, 35) of *N. benthamiana* strongly suggests that Chl Q_y_ to Zea S_1_ EET is a significant component of the qE response under excess light conditions.

## Results and Discussion

### CRISPR/Cas9 Mutagenesis of NPQ-Related Genes in *N. benthamiana*.

Although *npq* mutants of *Arabidopsis thaliana* were isolated by forward genetics previously (33), thylakoids of *Arabidopsis* have a limited qE capacity that makes it difficult to perform TA experiments with sufficient signal to noise. We employed a multiplexed CRISPR/Cas9 mutagenesis approach (36) to generate *N. benthamiana* mutants of NPQ-related genes, given its robust NPQ capacity. *N. benthamiana* orthologs of candidate NPQ genes (*NPQ4/PsbS*, *NPQ1/VDE*, and *LUT2*) were identified via BLAST (37, 38) using the allotetraploid *N. benthamiana* draft genome sequence v1.0.1 (Sol Genomics Network) (39) and the single-copy *Arabidopsis* protein sequences as queries. Gene structure was largely similar across paralogs, excluding *LUT2-2*, which was manually assembled by splicing two draft contigs in silico (*SI Appendix*, Fig. S1 and Table S1). The dual-paralog targeting guide RNA (gRNA) spacer sequences used for CRISPR mutagenesis are described in *SI Appendix*, Table S2. T_2_ homozygous knockout lines for PsbS, VDE, and LUT2 were screened from 5, 10, and 11 independent transformants, respectively. All isolated knockout lines, as well as the representative lines used in this study, are described in *SI Appendix*, Table S3.

### NPQ and Pigment Composition Phenotypes.

Differences in NPQ and pigment profiles between single and double paralog mutants revealed the relative contributions of each gene copy. Both copies of PsbS contribute additively to qE, with *PsbS1* acting as the dominant contributor (*SI Appendix*, Fig. S2). In contrast, *VDE1* is the sole paralog responsible for conversion of violaxanthin to zeaxanthin in *N. benthamiana* in response to high light (*SI Appendix*, Fig. S3). The two *LUT2* paralogs are functionally redundant, and loss of lutein requires knockout of both genes. The *N. benthamiana lut2-1 lut2-2* double mutant (hereafter *lut2*) maintains three times the xanthophyll cycle (VAZ) pool size relative to the wild type (WT), as has been observed in *Arabidopsis* (34), with a residual amount of Zea even after overnight dark acclimation (*SI Appendix*, Fig. S4).

**Fig. 1.**
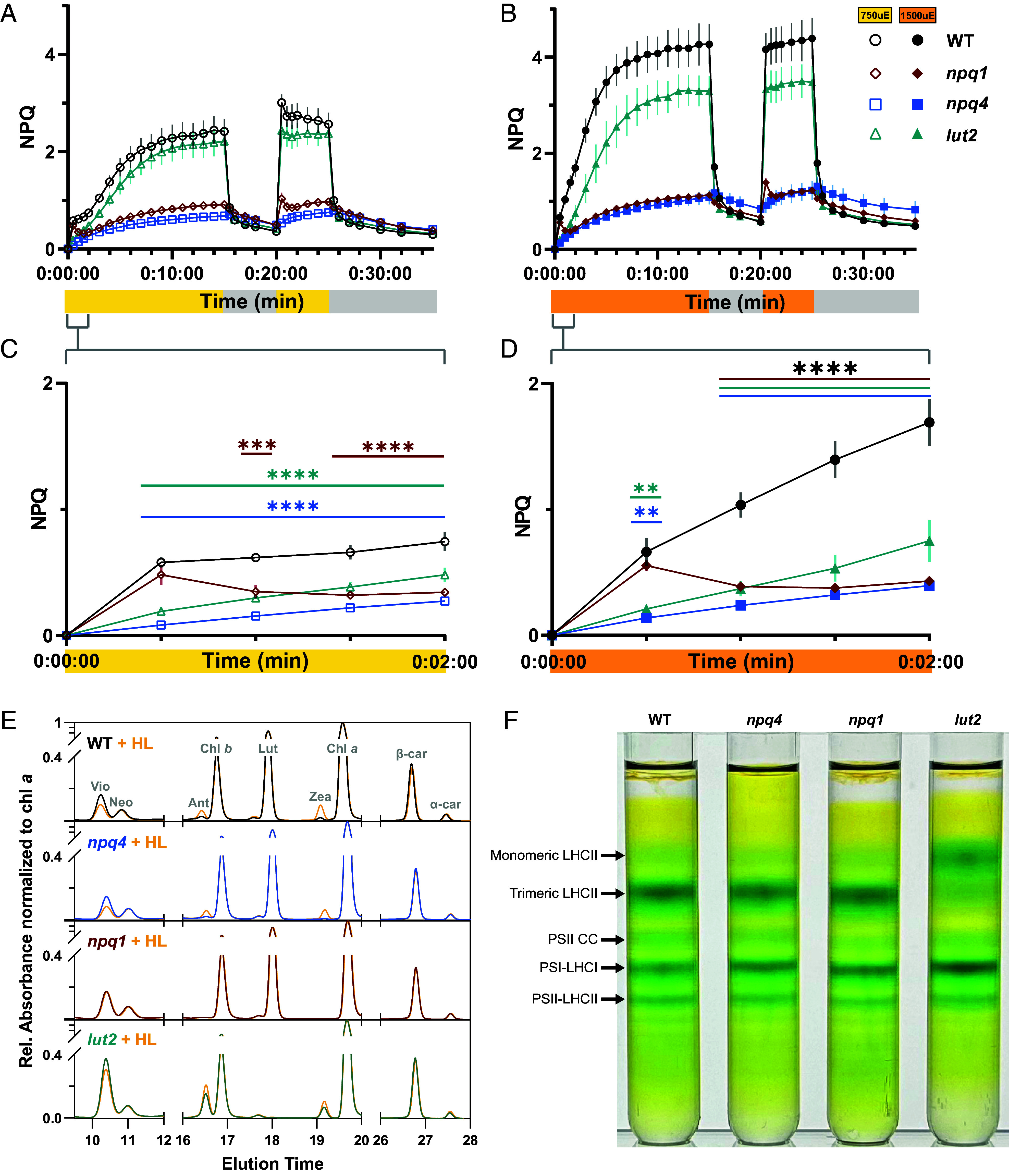
NPQ kinetics and thylakoid composition of tobacco mutants. NPQ kinetics of WT and homozygous knockout mutants under fluctuating actinic light at (*A*) 750 µmol photons m^−2^ s^−1^ (yellow bar) and (*B*) 1,500 µmol photons m^−2^ s^−1^ (orange bar). (*C* and *D*) NPQ of WT, *npq1*, *npq4*, and *lut2* in the first 2 min of actinic light. Data for n = 3 to 4 replicates each are shown as means ± SEM. WT (black circles), *npq1* (red diamonds), *npq4* (blue squares), and *lut2* (blue-green triangles). Open symbols are samples assayed at 750 µmol photons m^−2^ s^−1^. Closed symbols are samples assayed at 1,500 µmol photons m^−2^ s^−1^. Significance in (*C* and *D*) was determined by ordinary two-way ANOVA (α = 0.05) using Dunnett’s test for multiple comparisons against WT with significance denoted (**P ≤ 0.01, ***P ≤ 0.001, and ****P < 0.0001). (*E*) Representative chromatograms summarizing pigment profile of thylakoid extracts of each genotype before and after 15 min of high light (1,000 µmol photons m^−2^ s^−1^) normalized to chlorophyll *a*. (*F*) Separation of oligomeric photosynthetic complexes in dark-acclimated thylakoids by sucrose density gradient ultracentrifugation, with key bands denoted by arrows, in descending order: Monomeric LHCII, Trimeric LHCII, PSII Core Complex (CC), PSI-LHCI, and PSII-LHCII.

To assess how these mutations affected leaf-level NPQ, we used pulse-amplitude modulated fluorometry to measure NPQ at two actinic light intensities: 750 and 1,500 µmol photons m^−2^ s^−1^. As expected, knockout of *PsbS* (*psbs1 psbs2*, hereafter *npq4*) resulted in a loss of a vast majority of NPQ capacity, specifically qE, independent of light intensity (Fig. 1 *A* and *B*). The *lut2* mutant had indistinguishable NPQ from WT at 750 µmol photons m^−2^ s^−1^ (Fig. 1*A*), but significantly lower than WT NPQ at 1,500 µmol photons m^−2^ s^−1^ (Fig. 1*B*). Unlike *Arabidopsis* (33), loss of VDE (*vde1* or *vde1 vde2*, hereafter *npq1*) almost entirely abolished qE capacity, reaching near *npq4*-like levels (Fig. 1 *A* and *B*).

Closer inspection of the first 2 min of actinic light (Fig. 1 *C* and *D*) revealed additional differences in NPQ between the *npq4*, *npq1*, and *lut2 N. benthamiana* mutants. Under both light intensities, loss of PsbS or LUT2 resulted in a slower induction of NPQ that was indistinguishable between both genotypes. In contrast, NPQ in the *npq1* mutant had a transient increase in the first 30 s, reaching WT NPQ induction before declining to *npq4*-like levels after 1 min in high light. Increasing temporal resolution by measuring NPQ on a separate cohort of plants at staggered time intervals revealed a rapid transient increase in NPQ in the *npq1* mutant that was absent in *npq4* (*SI Appendix*, Fig. S5). Altogether, these data suggest that Lut is essential for a rapid but transient increase in NPQ upon dark–light transition and modestly contributes to NPQ at very high light intensities, but a vast majority of leaf-level NPQ in *N. benthamiana* is Zea-dependent.

### Thylakoid Composition of *N. benthamiana* NPQ Mutants.

Thylakoids were isolated from leaves of the characterized *N. benthamiana* NPQ mutants to test their functional relevance within our TA setup. Consistent with data from whole leaves and results reported from *Arabidopsis* mutants (6), thylakoids from WT and *npq4* leaves showed robust de-epoxidation of violaxanthin in response to HL. In contrast, *npq1* thylakoids showed no difference in pigment profile before and after the HL treatment. Thylakoids from *lut2* mutants had no detectable Lut, with a significantly increased VAZ pool size and persistence of Zea and Ant in dark-acclimated thylakoids (Fig. 1*E*) as observed in whole leaves (*SI Appendix*, Fig. S4). Sucrose density gradient ultracentrifugation of dark-acclimated thylakoids showed similar oligomeric supercomplex compositions in WT, *npq4*, and *npq1* thylakoids. In contrast, *lut2* mutant thylakoids showed a considerable loss of stable trimeric LHCII and a noticeable increase in the abundance of monomeric LHCII (Fig. 1*F*), also consistent with reports in *Arabidopsis* (15).

### Snapshot Fluorescence Lifetimes.

**Fig. 2.**
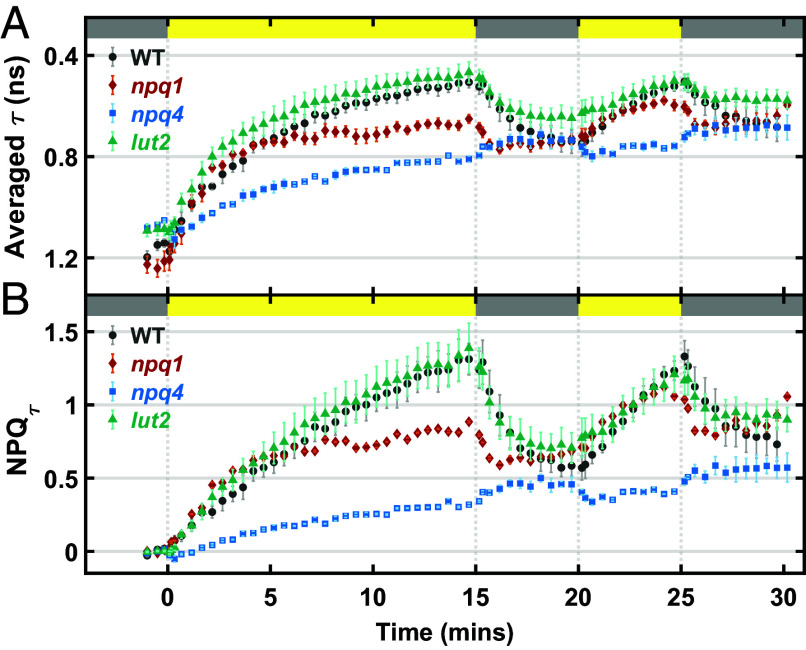
Snapshot chlorophyll fluorescence lifetime in *N. benthamiana* thylakoids. Periodic fluorescence lifetime data presented as a change in (*A*) averaged lifetime (τ) and (*B*) NPQ_τ_ values in response to 15–5–5–5 high light–dark sequence for WT (black dots), *npq1* (red diamond), *npq4* (blue square), and *lut2* (blue-green triangle) thylakoid membranes. Data for n = 3 to 4 replicates each are shown as means ± SEM. The duration of dark and high light (1,000 μmol photons m^−2^ s^−1^) exposures is indicated by gray and yellow bars, respectively.

We utilized snapshot fluorescence lifetime spectroscopy to explore the NPQ response of thylakoids under fluctuating light. In particular, the lifetime of excited state Chl (Chl*) was tracked in response to an alternating high light (1,000 μmol photons m^−2^ s^−1^) and dark sequence of 15–5–5–5 min by time-correlated single photon counting (TCSPC). Fig. 2*A* illustrates the changes in Chl* lifetime ($\tau_{\mathrm{avg}}$), which is calculated by taking an amplitude-weighted average of the two time constants obtained from a biexponential fitting of fluorescence decays measured at each sequence time, T. The degree of quenching of Chl* lifetime in response to light is quantitatively defined by a parameter, $NPQ_{\tau }\left(T\right)=\frac{\tau_{\mathrm{dark}}-\tau_{\mathrm{light}}\left(T\right)}{\tau_{light}\left(T\right)}$, where $\tau_{\mathrm{dark}}$ and $\tau_{\mathrm{light}}\left(T\right)$ are the average lifetimes under dark and high light exposure at the corresponding time, T, and hence, NPQ_τ_ reports the NPQ response. A rise in NPQ_τ_ indicates activation of the NPQ process and consequently, a quenching of Chl* lifetime.

In Fig. 2*B*, WT thylakoids exhibited a strong correlation between NPQ response and light exposure time, with NPQ_τ_ values reaching 1.4. As seen in leaves, thylakoids of the *npq4* mutant, which lack the pH-sensing protein PsbS (6), exhibited a complete loss of qE capacity. The slow, continuous rise of NPQ_τ_, which is most pronounced in *npq4* thylakoids, may reflect slower NPQ components, such as qZ or photodamage (40) which is activated on a minutes to hours long time scale. In contrast, *lut2* and *npq1* mutants showed some NPQ activity in response to high light but with differing NPQ capacities. While the observed biological variation in thylakoid samples, especially that of *lut2*, constrain our ability to make quantitative comparisons, the overall trends mirror NPQ phenotypes reported in Fig. 1 *A* and *B*. Relative to WT and *npq4*, the NPQ_τ_ level after 15 min of high light exposure follows the order: WT ≅ *lut2* > *npq1* > *npq4*.

### Snapshot TA Spectroscopy.

**Fig. 3.**
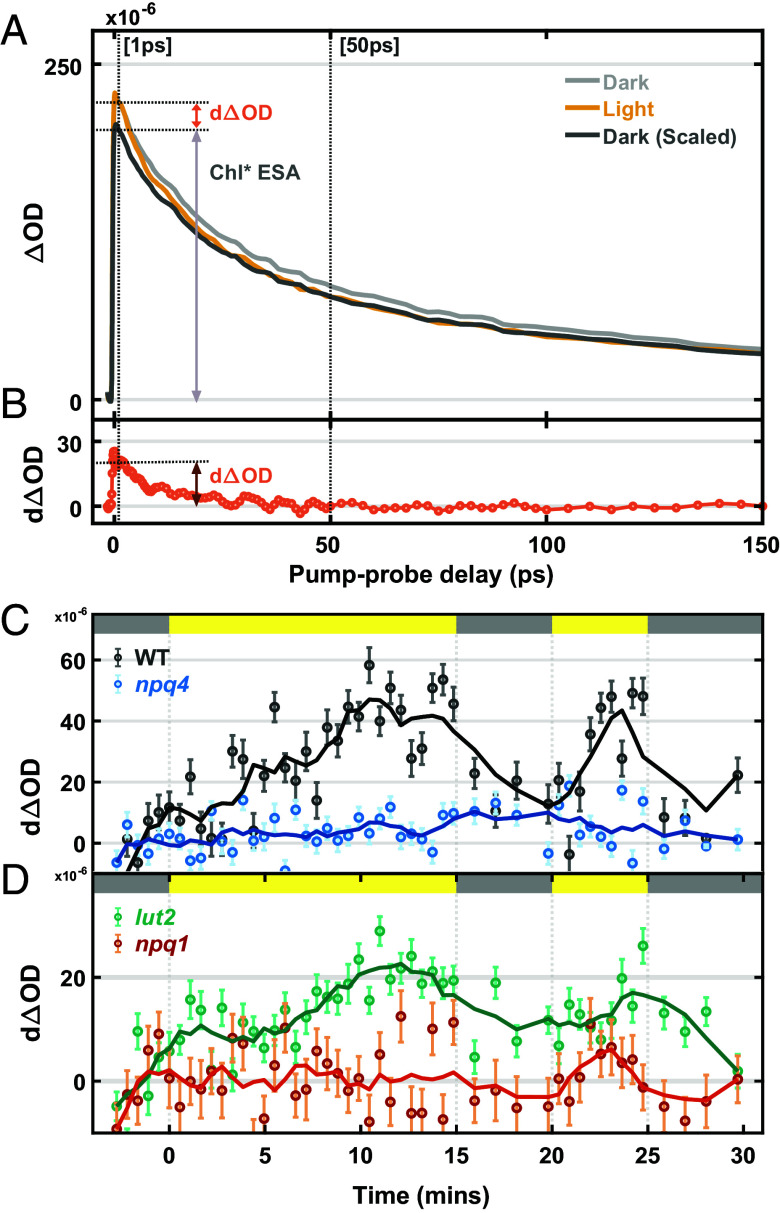
TA kinetic profiles and snapshot TA signals of *N. benthamiana* thylakoids. (*A*) The TA kinetic profiles of WT thylakoids scanned during initial dark (gray) and 15-min high light (yellow) cycles of a snapshot TA sequence. The black trace represents the scaled dark kinetic profile, which is normalized to the signal measured under high light at a 50 ps pump–probe delay. (*B*) The difference between the scaled dark and high light kinetic profile is shown by the orange trace. The difference at 1 ps pump–probe delay is defined as snapshot signal (*d*ΔOD). (*C* and *D*) Snapshot TA results presented as a change in excited state absorption signals (*d*ΔOD) probed at 540 nm in response to 15–5–5–5 high light–dark sequence for (*C*) WT (black dots), *npq4* (blue dots), (*D*) *lut2* (blue-green dots), and *npq1* (red dots) thylakoid membranes. The top bar indicates the dark (gray) and high light (yellow) cycles of the snapshot sequence. Each data point is shown as means ± SEM (n = 14). The solid lines are the smoothed results obtained by the moving average method.

Next, we applied TA spectroscopy to study EET from Chl* to Car, which has been proposed to play a key role in qE-type NPQ (18). The pump spectrum was centered at 675 nm to excite the Chl Q_y_ band. A continuum probe pulse was used to monitor the kinetics of the Car S_1_ state, which is not formed in one-photon absorption from the ground state but can be populated via EET from Chl*. Fig. 3*A* shows the TA kinetic profiles detected at 540 nm, measuring the carotenoid (Car) S_1_–S_n_ absorption, which overlaps with the broad S_1_–S_n_ absorption band of Chl* (35, 41). A difference in the TA kinetic profile between dark and high light conditions is expected when Chl* to Car energy transfer drives the qE response. The amplitude of TA signal is lower in high light due to significant quenching of Chl* by NPQ. The dark kinetic profile is further scaled by the high light TA signal at a 50 ps pump–probe delay, which is well beyond the reported Car S_1_ lifetimes (22, 42–44). This normalization ensures the matching of Chl* kinetics under both dark and high light conditions at longer times (>50 ps) and allows the extraction of the difference kinetic profile (Fig. 3*B*) from the scaled dark and high light profiles. The difference profile is expected to reflect Car S_1_ kinetics, and its amplitude should be correlated with the duration of the high light exposure.

Using the same actinic light sequence as the snapshot fluorescence lifetime measurements, we collected consecutive TA profile snapshots in a 30-s scanning window with an 18 nJ pump intensity. The averaged snapshot profile of the initial 3 min dark period is treated as the “dark kinetic profile” mentioned above. Then, each snapshot taken during the sequence is separately treated as a “high light profile” and subtracted from the scaled dark kinetic profile, which is similarly normalized to the TA signal of the current snapshot at 50 ps pump–probe delay (Fig. 3*A*). We define the snapshot TA signal (*d*ΔOD) as the 1 ps pump–probe delay amplitude of the difference kinetic profile (Fig. 3*B*) extracted from a snapshot. The snapshot TA results for WT thylakoids, shown in Fig. 3*C*, exhibit a pattern consistent with an NPQ response to high light (Fig. 2*B*). In contrast, the *npq4* mutant that lacks qE shows negligible change in snapshot TA signal between the high light and dark periods. For the carotenoid-related mutants, Fig. 3*D* shows a reasonable snapshot TA signal in *lut2*, which is correlated with the light-dependent NPQ response in a similar way to the WT. However, *npq1* lacks a robust snapshot TA response, much like *npq4*.

The above results show that the snapshot difference TA signal (*d*ΔOD) is correlated with the NPQ response. However, the origin of this difference remains ambiguous and controversial due to the high excitation pulse energy (29), which can lead to Chl*–Chl* EEA involving higher-order nonlinear kinetics and accelerating the decay. Therefore, instead of Car S_1_ to S_n_ absorption, Chl*–Chl* annihilation could result in differing kinetics under dark and high light conditions independent of NPQ. The amplitude of snapshot TA signal would then not accurately represent the extent of Chl*-to-Car S_1_ quenching. Contributions by EEA could also explain the differences in the magnitude of *d*ΔOD observed between WT and *lut2* (Fig. 3 *C* and *D*), likely due in part to differences in LHCII connectivity (Fig. 1*F*) and slowed energy transfer between Chls. Moreover, a closer look at the TA kinetic profiles shows that the decay under both dark and high light conditions is completed within 0.1 ns (Fig. 3*A*), which is much shorter than the Chl* fluorescence lifetime (0.3 to 1.4 ns) (Fig. 2*A*). Hence, it is evident that our TA kinetic profile includes a significant contribution from EEA, and it is clearly necessary to remove the EEA contribution to the TA signal before the Chl*-Car S_1_ energy transfer dynamics can be assessed.

### Annihilation-Free TA Spectroscopy.

To obtain a TA kinetic profile free from EEA dynamics, we employed a pump-intensity cycling-based high-order nonlinear signal separation method recently developed by Maly et al. (30). This perturbative method allows us to isolate the (2 N-1)th high-order nonlinear signal by measuring TA signals at N different pump intensities. For example, with N = 3, we can extract the pure 3rd-order nonlinear signal as well as higher-order (5th and 7th) nonlinear signals by taking a linear combination of TA pump–probe signals measured at three different pump intensities, *I*, 3*I*, and 4*I*, as follows:

[1] $$\begin{array}{c} \mathrm{PP}3\;I=2\mathrm{PP}\left(I\right)-\frac{2}{3}\mathrm{PP}\left(3I\right)+\frac{1}{4}\mathrm{PP}\left(4I\right)\\ \mathrm{PP}5\;I^{2}=-\frac{7}{6}\mathrm{PP}\left(I\right)+\frac{5}{6}\mathrm{PP}\left(3I\right)-\frac{1}{3}\mathrm{PP}\left(4I\right)\\ \;\\ \mathrm{PP}7\;I^{3}=\frac{1}{6}\mathrm{PP}\left(I\right)-\frac{1}{6}\mathrm{PP}\left(3I\right)+\frac{1}{12}\mathrm{PP}\left(4I\right), \end{array}$$

where PP is pump–probe signal measured at three different pump-intensities, *I*, 3*I,* and 4*I*. PP3, PP5, and PP7 represent the pure 3rd-, 5th-, and 7th-order nonlinear signals, respectively, at the corresponding lowest pump intensity *I*.

In this study, we measured the TA kinetics (*SI Appendix*, Fig. S6*A*) at pump pulse energies of 6 nJ (*I*), 18 nJ (3*I*), and 24 nJ (4*I*) (*SI Appendix*, Fig. S7). By using Eq. **1**, we extracted the pure nonlinear signals of order 3rd, 5th, and 7th, as illustrated in *SI Appendix*, Fig. S6*B*. These isolated signals correspond to the pure nonlinear signals obtained with the lowest pump intensity, *I*, i.e., 6 nJ. While the higher-order (5th and 7th) signals (PP5 and PP7) involve both single- and multiparticle dynamics, the isolated 3rd-order signal (PP3) represents single-molecule dynamics, such as Chl* relaxation, free from multiparticle annihilation dynamics. This decomposition is completely general, but interpretation of the different order dynamics, of course, depends on the system. When the exciton number dominates the multiparticle behavior, the EEA dynamics can be obtained from the 5th-order response. In the PP5 kinetic profile in *SI Appendix*, Fig. S6*B*, the negative component arises from EEA. The amplitude of the PP7 signal in *SI Appendix*, Fig. S6*B* is negligible, indicating that nonlinear signals beyond the 7th order can be ignored at a pump intensity of 6 nJ, which in turn, validates our perturbative treatment up to N = 3.

By separating higher-order nonlinear signals, the rapid decay component induced by EEA is removed from the TA kinetic profile, giving a slower decay in PP3 transient that represents Chl* relaxation. To validate the successful removal of EEA dynamics, we compared the isolated PP3 signal with the TA profiles measured with very low pump pulse energy (0.8 nJ). At such a low pump intensity range the TA signal becomes almost annihilation-free due to near-zero probability of Chl*–Chl* encounters. *SI Appendix*, Fig. S9 illustrates an excellent agreement between the extracted PP3 (6 nJ) and the low pump (0.8 nJ) intensity TA profile, confirming the successful separation of higher-order multiparticle annihilation dynamics and at the same time, achieving an excellent signal to noise ratio by using the pump-intensity cycling method described above.

**Fig. 4.**
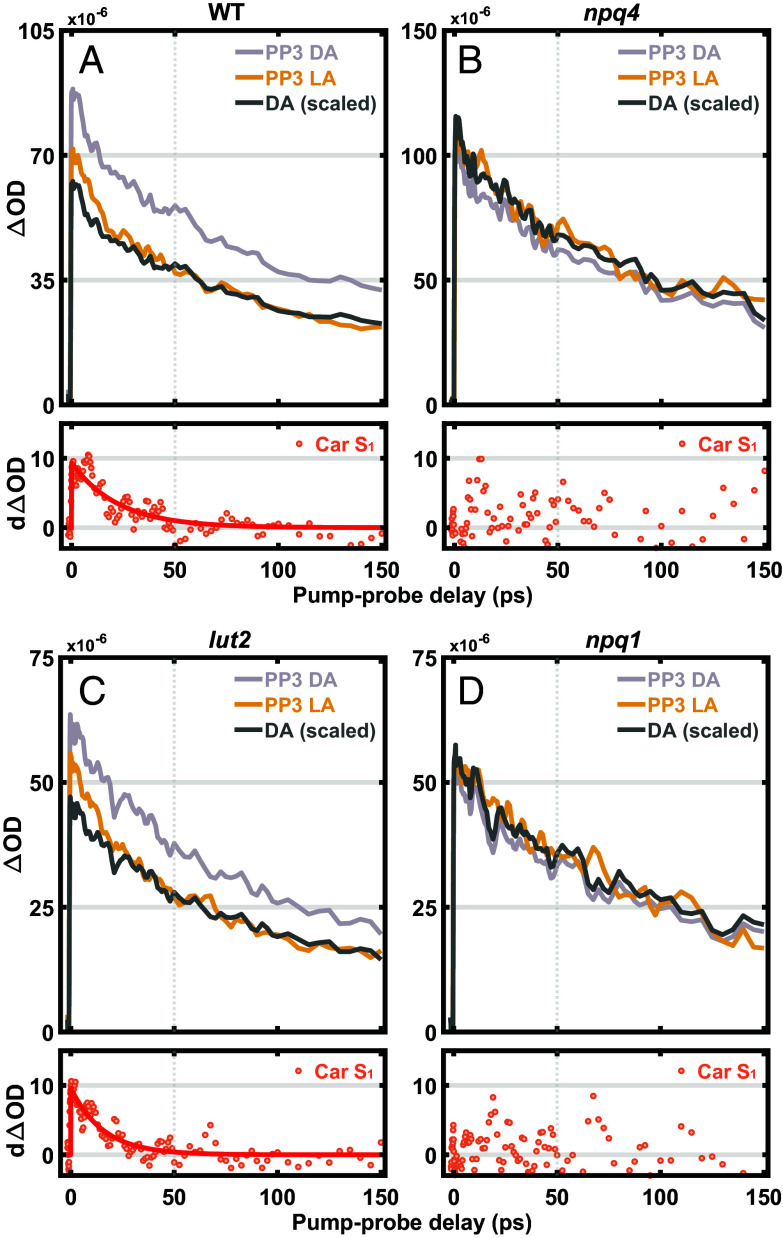
The PP3 kinetic profiles of *N. benthamiana* thylakoids. Pure TA kinetic profiles of (*A*) WT and (*B*) *npq4,* (*C*) *lut2,* and (*D*) *npq1* thylakoid membranes under dark (gray) and light-acclimated (yellow) conditions. The black trace in each panel represents the scaled dark-acclimated pump–probe kinetic profile normalized to the corresponding light-acclimated transient averaged over the pump–probe delay of 50 to 100 ps. The red dotted traces were obtained by subtracting scaled dark-acclimated from light-acclimated PP3 kinetic profile, corresponding to the evolution of the Car S_1_ population. The red line in (*A*) and (*C*) is a monoexponential fit.

The pump-intensity cycling-based TA measurements were performed under both dark and high light conditions, and annihilation-free PP3 kinetic profiles were extracted (Fig. 4). The dark PP3 profiles are scaled by normalizing to the high light PP3 signal at a 50 to 100 ps pump–probe delay. The difference in the PP3 kinetic profiles (Fig. 4 *A*–*D*, *Bottom* panel) was obtained by subtracting the scaled dark PP3 profile from the high light PP3 profile, which shows a nonzero difference PP3 signal in WT. Thus, by utilizing the high-order signal separation method, we are now able to confirm that this observed difference in PP3 signal decay in WT originates from Car S_1_, and hence, it provides direct and unambiguous evidence of Chl* to Car EET during qE. Furthermore, the difference PP3 signal shows a monoexponential decay with a time constant of ~22 ps, which is longer than the typical lifetime (8 to 16 ps) of different carotenoids reported previously (42). The decay time of the Car S_1_ signal should not be equated with the solution lifetime of Zea (or Lut), because the decay is convoluted with the range of timescales for excitation to reach the site of Chl–Car interaction. Numerical calculations (*SI Appendix*, Fig. S10 and *SI Method*) clearly show that this convolution lengthens the decay of the Car S_1_ signal compared to the intrinsic S_1_ lifetime. Thus, the fitted decay time of 22 ps is completely compatible with the measured S_1_ lifetime of Zea of 8 to 10 ps observed in vitro (42).

We compared the WT and the *npq4* mutant to investigate whether the presence of Car TA signal is related to qE activity. In contrast to WT, the *npq4* mutant (Fig. 4 *A* vs. *B*) shows no difference in the PP3 kinetic profile under dark and high light conditions and thus, indicates an absence of Chl* to Car EET. Overall, these results suggest a strong correlation between pH-sensing via protonation of PsbS, Chl–Car EET, and qE activity.

### The Relative Contribution of Zeaxanthin and Lutein.

Although the difference TA signal measured with the annihilation-free method clearly shows the Car S_1_ dynamics, it is still not clear from these data which specific carotenoid plays the central role in qE activity. Zea and Lut have both been proposed to be involved in the quenching process of qE (9). As mentioned above, distinguishing between Zea and Lut based on the decay time of Car S_1_ is not feasible. It is also difficult to distinguish Zea and Lut in TA measurements because the S_1_–S_n_ absorption of both appears at a very similar wavelength (Zea 540 nm; Lut 530 nm). To resolve the contributions of each carotenoid, we carried out the snapshot fluorescence lifetime, snapshot TA, and annihilation-free TA, for the two carotenoid-related *N. benthamiana* mutants, *lut2* and *npq1*.

In the *lut2* mutant, the production of Lut is knocked out, and the VAZ pool of xanthophylls is increased (Fig. 1*E*), leaving Zea as the most likely source of the Car S_1_ signal. The qE and snapshot TA Chl*-Car EET response (Figs. 2 and 3*D*) to high light were clearly observed in the *lut2* mutant. Furthermore, annihilation-free TA data show a nonzero differential (*d*ΔOD) PP3 signal (Fig. 4*C*) similar to WT (Fig. 4*A*), indicating that the absence of Lut does not significantly affect the formation of Car S_1_. In contrast, *npq1*, which lacks Zea, shows neither a snapshot TA signal (Fig. 3*D*) nor a differential PP3 signal (Fig. 4*D*) much like *npq4* (Fig. 4*B*), despite exhibiting a significant non-Zea-dependent qE response under high light.

The specific role of Zea in quenching has been long debated (11, 45). Zea was proposed by Horton and coworkers (21) to act indirectly and allosterically by aiding the aggregation of LHCII complexes, leading to quenching of Chl* lifetime. Later studies found that Zea can also act as a direct quencher through the charge transfer (CT) mechanism, which involves the formation of charge-separation states (Chl^·−^ − Zea^·+^) (13, 41, 46), or by Chl*-Zea EET. Both processes were observed by Park et al. in snapshot TA measurements of spinach thylakoid membranes (23) and lutein-less *N. oceanica* (17, 18). However, these prior observations were clearly perturbed by EEA. Our annihilation-free TA results on the WT and the *lut2* mutant show that the Zea S_1_–S_n_ signal is correlated with high light exposure time. These results strongly suggest that Zea can act as a direct quencher via an EET mechanism and is likely a significant quenching route in qE. In contrast, in the *npq1* mutant (lacking Zea), a smaller but noticeable NPQ response suggests that Lut acts as a less effective quencher. The absence of both a PP3 difference signal and a snapshot TA signal in *npq1* suggests that Chl*-Lut EET is not a major route of quenching relative to the contributions of Zea EET following sufficient VDE activity. However, Lut may still act as a direct quencher through a CT mechanism as reported earlier in vitro (14, 47).

Unlike Zea, the concentration of Lut does not fluctuate during periodic light exposure, and the production of Lut in *npq1* is similar to that in WT (Fig. 1*E*). In LHCII, Lut has been suggested from theoretical studies to exhibit charge-transfer-mediated quenching in the absence of Zea (48). However, the decrease in NPQ activity of *npq1* most likely results from the absence of Zea, which, on a per molecule basis, is a more effective quencher than Lut (49).

It is important to conceptually reconcile the similar maximum NPQ capacities seen in WT and *lut2* leaves (Fig. 1*B*) and thylakoids (Fig. 2*B*). Earlier, we had noted an increase in the VAZ pool size in *lut2* and an associated small de-epoxidized xanthophyll population (Ant and Zea) after overnight dark acclimation (*SI Appendix*, Fig. S4). A larger de-epoxidized VAZ pool might be expected to provide additional quenching under high light conditions. However, in *Arabidopsis* and *N. benthamiana*, loss of Lut disrupts trimer stability of the major light-harvesting complex LHCII (Fig. 1*F*) (15). Major LHCII and monomeric PSII minor antennas CP24, CP26, and CP29 bind carotenoids and are thought to play a significant role in quenching excitation energy. In the LHC proteins, Lut typically occupies the L1/L2 sites, while Zea, following the action of VDE, has been found to bind the L2 site when reconstituted in vitro (50). However, recent spectroscopic data suggest that in vivo, Zea is unable to bind at these high Lut-affinity sites and may bind and act at the periphery of LHCs instead (5, 45). In the *lut2* mutant, it is possible that the excess Vio can replace Lut and occupy the L1 site (15), or it may stochastically bind and retain free Zea during LHC protein folding and assembly, given that the L1 site is reported to be less accessible for VDE or ZEP activity (51). We expect the de-epoxidation of Vio to be slower in the internal L1/L2 sites typically bound by Lut (52). This is in agreement with our fluorescence lifetime data, where Chl* has a slightly shorter lifetime (1.09 ± 0.02 ns) in *lut2* than in WT (1.16 ± 0.02 ns) in dark-acclimated thylakoids (Fig. 2*A*), possibly indicating a contribution to NPQ from preexisting Zea in the *lut2* mutant (Fig. 1*E*). Following 15 min of high light exposure, Figs. 2 and 4 show that both the quenching capacity and Car S_1_ signal are very similar to that of the WT, and both *lut2* and WT show similar magnitudes of quenched fluorescence lifetime (0.49 ± 0.02 ns). The results suggest that despite the likely presence of some Vio/Zea in the L1 and L2 sites of LHC proteins in the *lut2* mutant, Zea in this site is either not or only weakly involved in EET quenching.

### Chl*–Chl* Annihilation Dynamics and the Exciton Diffusion Length.

**Fig. 5.**
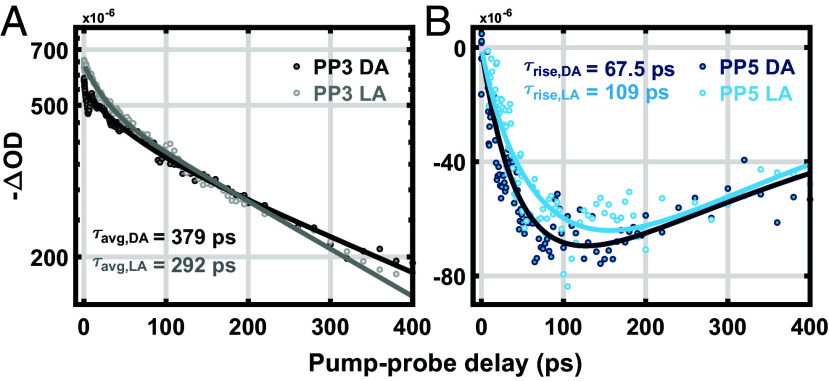
Ground-state bleach kinetic profiles of WT thylakoids. The pure (*A*) PP3 and (*B*) PP5 ground-state bleach kinetic profiles of WT thylakoid membranes under dark (DA, dark dots) and light-acclimated (LA, light dots) conditions. The solid lines in PP3 and PP5 are fit profiles with biexponential decay and 3D diffusion model, respectively.

Bennett et al. (53) proposed that qE within the thylakoid membrane could be characterized by a single quantity: the exciton diffusion length, *L_D_*. *L_D_* is defined as the distance an exciton travels when the excitation probability has decayed to 1/e of its initial value. Our pump-intensity cycled TA measurement provides a unique noninvasive way to directly evaluate this quantity under NPQ conditions in thylakoids. In particular, the isolated 5th-order (PP5) transient signal involves two-exciton annihilation dynamics, and the change in these dynamics from dark-acclimated to high light conditions can serve as a proxy for calculating the change in exciton diffusion length during energy-dependent quenching (qE). Fig. 5 compares the isolated PP3 and PP5 kinetic traces measured under dark and high light probed at 680 nm in WT, where the ground-state bleach of Chl is maximized. The Chl* lifetime quenching under high light results in a faster (292 ps) decay of the PP3 signal compared to that (379 ps) in the dark (Fig. 5*A*). The rise of the PP5 signal, on the other hand, reports the rate of annihilation, which is faster (t_rise_ = 68 ps) under high light compared to that (t_rise_ = 109 ps) in the dark as illustrated in Fig. 5*B*. The diffusion length can be quantitatively evaluated from the annihilation rate using a diffusion-limited kinetic model and the exciton lifetime. Assuming the excitons migrate within a three-dimensional energetic network formed by pigments, and that excitons instantly quench when they encounter each other within a 2 nm radius, we estimate the diffusion length in dark-acclimated (NPQ off) and high light (NPQ on) cases to be 62 ± 6 and 43 ± 3 nm, respectively (*SI Appendix*, Table S5). Thus, our data show that the diffusion length decreases by nearly 31% under high light compared to that in the dark-acclimated case. These values are similar to the values proposed by Bennett et al. (53) from their multiscale model of 50 nm (dark-acclimated) and 25 nm at an NPQ value of 2.5, which is somewhat higher than that for the WT thylakoids studied here. The calculated *L_D_* values of the mutants show a good correlation with NPQ capacity, which will be discussed in future work.

### Concluding Remarks.

There has been extensive debate on the roles and molecular mechanism(s) in which Lut and Zea contribute to NPQ (9–21). The combined snapshot and annihilation-free TA data presented here strongly imply the direct involvement of Chl* to Car (Zea) S_1_ EET as an important component of energy-dependent quenching (qE). We were unable to detect a transient signal from Lut in the *npq1* mutant, lacking Zea, and in earlier work on WT spinach thylakoids (23) despite ample evidence that Lut does contribute to qE, especially the initial response. It is possible that the 510 nm shoulder reported by Mascoli et al. in isolated CP29 (54) is below our detection limit in thylakoids. We also cannot exclude possible contributions of a charge transfer-type quenching to qE by Lut and/or Zea. Such possibilities remain to be investigated by exciton annihilation-free studies of the type described here. Our initial analysis of the 5th-order response supports the suggestion of Bennett et al. (53) that qE capacity can be defined by a single physical parameter, the exciton diffusion length, *L_D._*

## Materials and Methods

### Plant Material and Growth Conditions.

Transgenic *N. benthamiana* (accession *Nb-1*) lines were generated via *Agrobacterium*-mediated transformation by the Ralph M. Parsons Foundation Plant Transformation Facility at UC Davis (https://ptf.ucdavis.edu/). *N. benthamiana* plants were grown with a 10-h daylength in a south-facing greenhouse. Seeds were germinated directly on a mixture of four parts Sunshine Mix #1 (Sungro) and one part perlite. Plants were fertilized with JR Peter’s Blue 20–20–20 fertilizer monthly.

### Construct Cloning and gRNA Design.

Candidate gRNAs were identified using CRISPR-P (55), and two high-scoring gRNAs for each gene were chosen depending 1) on their ability to target both *N. benthamiana* orthologs and 2) sequence similarity to the orthologous *Arabidopsis* gene downstream of the predicted chloroplast transit peptide sequence. Each set of two gRNAs was synthesized as a gBlock (Integrated DNA Technologies), interspersed with a gRNA scaffold and tRNA linker to allow for polycistronic gRNA expression as previously described (36). The insert was cloned into a modified pCAMBIA2300 backbone containing a dual 35S promoter driving SpCas9 (56) and an *Arabidopsis* U6-26 promoter driving expression of gRNAs (57) prior to stable transformation.

### Chlorophyll Fluorescence Phenotyping of NPQ in Leaves.

CRISPR/Cas9 knockouts of target orthologs were identified by whole plant and/or leaf punch phenotyping of chlorophyll fluorescence at room temperature using an Imaging-PAM Maxi (Walz) pulse-amplitude modulation fluorometer. Differences in NPQ between genotypes were quantified on overnight dark-acclimated plants using an FMS2+ fluorometer (Hansatech Instruments Ltd.). Fluorescence yield measurements in the dark (F_o_, F_m_) and after actinic light exposure (F_o_′, F_m_′) were measured during a sequence of 15 min high light, 5 min darkness, 5 min high light, 10 min darkness at two white light intensities: 750 µmol photons m^−2^ s^−1^ and 1,500 µmol photons m^−2^ s^−1^ light. In both instances, NPQ was calculated as:

$$\mathrm{NPQ}=\frac{F_{m}-F_{m}\prime }{F_{m}\prime }.$$

### Genotyping of CRISPR/Cas9 Edits.

Genomic DNA from putative knockout lines was isolated and genotyped by Phire Plant Direct PCR Master Mix (ThermoScientific™, Catalog #F160L) using the supplied dilution buffer. DNA was amplified by PCR using primers that spanned the two gRNA target sites for each gene of interest, with primer pairs specific to one of the two highly similar paralogs (*SI Appendix*, Table S4). PCR products were purified and sequenced by Sanger sequencing. Segregating gene-edited mutations were identified in the T_0_ population via SangerTrace analysis (ice.synthego.com) (58), and promising knockout candidates were analyzed for stable, heritable phenotypes and genotypes in the T_1_ generation. Segregation of the Cas9 transgene was determined via changes in chlorophyll fluorescence after antibiotic treatment as previously described (59).

### High-Performance Liquid Chromatography of Leaf Extracts.

Total leaf chlorophylls and carotenoids (neoxanthin, violaxanthin, antheraxanthin, lutein, chlorophyll *b*, zeaxanthin, chlorophyll *a*, and β-carotene) were analyzed by high-performance liquid chromatography (1100 HPLC, Agilent) using a C18 column (ODS1, 5 µm, Waters) (60) or a C30 column (YMC Carotenoid, 5 µm, YMC America) (61) and quantified against a dilution series of standards. Briefly, leaf tissue, either dark acclimated or exposed to high light, was flash frozen in liquid nitrogen and ground in tubes containing Lysing Matrix D beads using a FastPrep-24 5G™ High-Speed Homogenizer (6.0 m/s 1 × 40 s, MP Biomedical). Pigments were extracted twice in 150 µL 100% ethanol until the remaining leaf debris was white in color. Samples were extracted on ice and in the dark to minimize pigment degradation and evaporation of the solvent.

### Isolation of Thylakoid Membranes.

Five-week-old *N. benthamiana* plants were dark acclimated for 1 h, after which whole leaves were sampled and stored in moist paper towels wrapped in foil at 4 °C overnight. Isolation of crude thylakoid membranes was performed in a dark cold room (4 °C) using the protocol described by Gilmore et al. (62) with the following changes. Chilled leaves (~10 g) were blended in a grinding buffer composed of 0.33 M sorbitol in place of 0.33 M dextrose, 0.2% L-ascorbic acid in place of 0.2% sodium ascorbate, and with the addition of 10 mM EDTA, adjusted to a final pH of 8.2. Leaves were blended as described, gravity filtered through four layers of miracloth, and centrifuged briefly for 2 min at 2,000×*g* to pellet starch that otherwise contributed to high signal scattering. The supernatant was carefully poured into fresh tubes and centrifuged for 10 min at 2,000×*g*, and the resulting pellet was gently resuspended by paintbrush in the described buffer A while avoiding any residual starch. Chlorophyll was quantified using 80% acetone as described by Porra et al. (63), and thylakoid samples were adjusted to 75 µg Chl/mL in reaction buffer immediately before measurements. The reaction buffer (pH 8) contained 30 mM L-ascorbic acid, 0.5 mM ATP, and 50 μM methyl viologen.

### Sucrose Gradient Ultracentrifugation.

Isolated thylakoid membranes were washed with 25 mM HEPES-NaOH (pH 7.6) at 15,000×*g* for 10 min at 4 °C. The pellet was resuspended with the same buffer at 0.5 mg Chl/mL and solubilized with 1% (w/v) n-dodecyl-α-D-maltoside (Anatrace) for 30 min on ice. The unsolubilized fraction was removed by centrifugation at 21,000×*g* for 5 min at 4 °C. The solubilized thylakoid membrane fractions were loaded onto sucrose gradients (0.1 to 1.3 M sucrose with 25 mM HEPES-NaOH (pH 7.6) and 0.03% (w/v) n-dodecyl-α-D-maltoside) and centrifuged at 154,300×*g* (SW 41 Ti rotor, Beckman Coulter) for 24 h at 4 °C.

### Fluorescence Lifetime Snapshot Measurements.

TCSPC was used to measure changes in Chl fluorescence lifetimes of the thylakoid samples during high-light exposure and the subsequent dark periods, as previously described (64). A Ti:sapphire oscillator (Coherent, Mira900f, 76 MHz) generated pulses at ~808 nm which were frequency-doubled to ~404 nm by a beta barium borate crystal and used to excite the Soret band of Chl *a*. With a beam splitter, part of the excitation beam was divided to a photodiode (Becker-Hickl, PHD-400) to provide SYNC signals. The remainder of the excitation beam was then incident at an approximately 70° angle to the cuvette surface with its power set to 1.0 mW, saturating the reaction centers. During measurements, the samples were exposed to an actinic light (Leica KL1500 LCD) sequence, composed of alternating high-light (1,000 µmol photons m^−2^ s^−1^) and dark periods of 15–5–5–5 min. Fluorescence emission was collected by a microchannel plate (MCP)-photomultiplier tube (PMT) detector (Hamamatsu R3809U MCP-PMT) after a monochromator (HORIBA Jobin-Yvon; H-20), which was set to 680 nm to detect Chl a Qy band fluorescence. The excitation, actinic light, and detection were coordinated by a series of shutters controlled by a LabVIEW program. Each snapshot was measured at intervals of 30 s. Each fluorescence decay profile over 10 ns was fitted with a biexponential decay function and the amplitude-weighted average lifetime was calculated as:

$$\tau =\frac{\sum_{i}A_{i}\tau_{i}}{\sum_{i}A_{i}},$$

where $A_{i}$ and $\tau_{i}$ are the amplitudes and fluorescence lifetimes of the ith fitting component, respectively. The NPQ capacity is defined by ${\mathrm{NPQ}}_{\tau }(T)=\frac{\tau_{\mathrm{dark}}(T=0)-\tau (T)}{\tau (T)}$, where $\tau_{\mathrm{dark}}\left(T=0\right)$ is the average of amplitude-weighted average lifetimes of the three initial dark snapshots, and τ(T) is amplitude-weighted average lifetime at the corresponding snapshot sequence time *T*.

### Snapshot TA.

The snapshot TA measurement was similar to previous work (18), which combines the pump–probe TA spectroscopy with a sequenced external actinic light source. The pump–probe TA system used a regenerative amplifier (RegA 9050, Coherent) seeded by Ti/sapphire Laser (Vitara-T, Coherent) to generate mode-locked 800 nm laser pulses at 250 kHz repetition rate. The pulse was modulated by an external stretcher/compressor and then split into pump and probe beam path by a beam splitter. The pump pulse was centered at 675 nm (FWHM 35 nm) with an optical parametric amplifier (OPA, Coherent) and compressed by prisms to a FWHM of an autocorrelation trace of ~49 fs. The pump intensity was adjusted between 0.8 to 32 nJ by a neutral-density filter wheel. For the probe beam path, a visible continuum was generated by a 1 mm sapphire crystal and filtered by a 700-nm short-pass filter. The pump and probe pulses passed through a 0.5 mm thick cuvette and were overlapped on a sample at the magic-angle ($54.7^{\circ }$) polarization. The pump–probe cross-correlation time was ~100 fs at 540 nm, and the diameter of pump and probe pulses at the sample position was 160 and 80 μm, respectively. To prevent continuous excitation of a single spot, the cuvette was vibrated at 7 Hz in a direction perpendicular to the probe beam path. After passing through the sample, the probe beam was filtered by a polarizer to remove scattered pump light. A monochromator (SpectraPro 300i, Acton Research Corp.) was used to select probe wavelength (540 nm for excited state absorption; 680 nm for ground state bleach). The exit pulses were collected by a diode detector (DET10A, Thorlabs), generating analog signal input to a lock-in amplifier (SR830, Stanford Research) which synchronized the pump–probe signals with a chopper positioned in the pump beam path.

In snapshot TA measurements, we controlled an external actinic light during a 10–5–5–5-min sequence of alternating dark and high light (1,000 µmol photons m^−2^ s^−1^) periods. Each TA profile was collected in a 30-s scanning window at intervals ranging from 3 to 69 s with 18 nJ pump intensity. A shutter was positioned in front of the sample to block the pump and probe pulses during the intervals. After the snapshot sequence, the snapshot TA signal was evaluated by:

$$d\Delta \mathrm{OD}(T)\;=\;\Delta {\mathrm{OD}}_{1ps}(T)\;-\;\Delta {\text{OD}}_{1ps}\left(\mathrm{Dark}\right)\;\times \left(\frac{\Delta {\text{OD}}_{50ps}\left(T\right)}{\Delta {\text{OD}}_{50ps}\left(\mathrm{Dark}\right)}\right),$$

where $d\Delta OD\left(T\right)$ is the snapshot TA signal at corresponding sequence time T. $\Delta OD\left(Dark\right)$ and $\Delta OD\left(T\right)$ are the TA signal during the initial dark and at sequence time T, respectively. The subscript of dΔOD presents the corresponding pump–probe delay time.

### EEA-Free Transient Spectroscopy.

EEA-free transient spectroscopy applied a similar pump–probe TA setup as described above. To obtain a set of separated high-order TA signals (PP3, PP5, and PP7), we collected TA profiles with 6, 18, and 24 nJ pump intensity as for a complete set of intensity-cycling-based measurements. Each set of measurements was conducted from the lowest to the highest pump intensity. The pump intensities were monitored by a power meter (PM100D, Thorlabs) before and after collecting a TA profile to ensure a consistent intensity during the measurement. The dark condition profiles were measured in a dark room with thylakoid samples dark-acclimated for more than 30 min. Following dark condition measurements, the high light condition profiles were measured after 15 min actinic light exposure at 1,000 μmol photons m^−2^ s^−1^. Each set of intensity-cycling-based measurements was completed within 20 min, and the TA measurement of each thylakoid sample was completed within an hour to preserve the NPQ activity of the thylakoids.

*SI Appendix*, Fig. S7 shows the TA profiles for each intensity-cycling-based measurement. The signal amplitude dependence on pump intensities is shown in *SI Appendix*, Fig. S8, where we selected TA signals at 1 ps pump–probe delay time to represent the amplitude. This dependency typically shows a nonlinear relationship due to the involvement of higher-order nonlinear signals. However, the separate high-order signals (PP5 and PP7 in *SI Appendix*, Fig. S6*B*) have amplitudes that are much smaller than that of PP3 at 1 ps delay time. Since the signals at 1 ps are dominated by PP3, we expected them to show a dependence on pump intensity, which is in line with the results in *SI Appendix*, Fig. S8.

To confirm that the kinetics of the extracted PP3 profile are consistent with annihilation-free kinetics, we measured the TA profile at 0.8 nJ pump intensity as a reference to reduce the influence of the higher-order nonlinear signals. In *SI Appendix*, Fig. S9, the PP3 signals showed a slower decay than the profiles measured above 6 nJ, indicating that the extent of the high-order signals are reduced. The PP3 signal (6 nJ) also matched the profile of 0.8 nJ. Moreover, compared to the measurement at 0.8 nJ, the PP3 results demonstrate that the high-order nonlinear signal separation method provides an excellent signal-to-noise ratio and requires a much shorter experiment time.

### Probing Exciton Diffusion with EEA Dynamics.

By extending the higher-order nonlinear signal separation method by Maly et al. (30), we developed a procedure to probe the exciton diffusion behaviors with 5th-order nonlinear TA signals (PP5) which involves the EEA dynamics:

$${\text{Chl}}^{*}+{\text{Chl}}^{*}\overset{\gamma }{\to }{\text{Chl}}^{*}+\text{Chl},$$

where γ is the annihilation rate constant.

In a vast energetic network system like thylakoid membranes, exciton migration can be considered a diffusion process (28). Moreover, given the large number of pathways in the network, the diffusion of excitons is expected to serve as the bottleneck for Chl*–Chl* encounters. EEA can be approached as a diffusion-limit reaction, and the annihilation rate constant γ is approximated to be equal to the rate constant k of the diffusion reaction. This k is directly related to the diffusion constant and diffusion behavior of excitons and can be obtained by analyzing the PP5 kinetics. According to Maly’s excitonic model (30), the response function of PP5 for ground state beach signals is written as follows:

$$\text{PP}5\left(t\right)=A\left(1-e^{-kt}\right)\times Decay(t),$$

where A is a preexponential constant. Decay(t) contains the single-particle dynamics, such as Chl* relaxation, and is replaced by the fitted function of the PP3 profile in a biexponential decay form. The k is a rise time constant, corresponding to the annihilation rate constant γ, or diffusion rate constant under our approach.

To convert the rate constant k into diffusion-related parameters, we made further assumptions: 1) the excitons travel in a three-dimensional membrane with 4 nm thickness. 2) Two excitons can be found in a mean area with a 15 nm radius according to excitation density. 3) EEA occurs immediately when two excitons encounter each other within a 20 Å reaction radius. The diffusion constant (D) is obtained by D=k/4πR, where R is the reaction radius, and k is the diffusion-limited reaction rate constant. k is normalized by the mean volume of a two-exciton system. The diffusion length ($L_{D}$) of excitons is derived from the diffusion constant by $L_{D}=\sqrt{6D\tau }$, where τ is the averaged Chl* lifetime from PP3 profile fitting.

*SI Appendix*, Table S5 lists the calculated diffusion-related parameters for WT thylakoids under dark and high light conditions. Our $L_{D}$ results are similar to the predicted values from the multiscale model proposed by Bennett et al. (53) of 50 nm under dark (NPQ = 0) and 31 nm under high light (NPQ = 1.5) conditions, showing an inverse relationship between $L_{D}$ and NPQ. Although altering our assumptions regarding the reaction radius or dimensionality will affect $L_{D},L_{D}$ will still exhibit an inverse dependence on the NPQ value. The ratio ($L_{D,Light}/L_{D,Dark}$) is independent of the reaction radius or system size. Moreover, replacing the dimensionality with a 1D or fractal dimension system still shows a reduced $L_{D}$ under high light. Our results demonstrate the potential of the higher-order nonlinear signal separation method and the possibility of probing the change of the exciton diffusion behavior in photosynthetic organisms.

## Supplementary Material

Appendix 01 (PDF)

Dataset S01 (XLSX)

Dataset S02 (XLSX)

Dataset S03 (XLSX)

Dataset S04 (XLSX)

Dataset S05 (XLSX)

Dataset S06 (XLSX)

Code S01 (TXT)

Code S02 (TXT)

Code S03 (XLSX)

Code S04 (XLSX)

## Data Availability

All raw data used to generate the figures and codes of model analysis within this work are included in Datasets S1–S6 and Codes S1–S4, respectively. All other data are included in the manuscript and/or supporting information.
