# Supplementary material for: Chlorophyll to zeaxanthin energy transfer in nonphotochemical quenching: An exciton annihilation-free transient absorption study
Source: Proc Natl Acad Sci U S A. 2024 Oct 8;121(42):e2411620121. doi: 10.1073/pnas.2411620121 (PMC11494355; doi:10.1073/pnas.2411620121)
Supplement: Supplementary file 1 — Appendix 01 (PDF) [file pnas.2411620121.sapp.pdf]

PNAS

Supporting Information for
Chlorophyll to Zeaxanthin Energy Transfer in Non-Photochemical Quenching: An Exciton Annihilation-free Transient Absorption Study

Tsung-Yen Lee, Lam Lam, Dhruv Patel-Tupper, Partha Pratim Roy, Sophia A. Ma, Henry E. Lam, Aviva Lucas-DeMott, Nicholas G. Karavolias, Masakazu Iwai, Krishna K. Niyogi and Graham R. Fleming

Krishna K. Niyogi and Graham R. Fleming
Email: grfleming@lbl.gov and niyogi@berkeley.edu

This PDF file includes:

Supporting text
Figures S1 to S10
Tables S1 to S5
SI References

SI Method

Convolution Analysis for Carotenoid Lifetime

As noted in the main text, we obtain a longer Car S_1 lifetime in our TA results than previously reported values[1]. The kinetic profile of Car S_1 in thylakoid membranes cannot be described by the rise-decay kinetics of a single species. Instead, a convoluted kinetics model is required. Only if the initially excited Chl molecule directly transferred excitation very rapidly to the carotenoid can the formation of the Car S_1 signal be modeled by an instrument function- limited one followed by the solution S_1 decay profile. In practice there will be a distribution of arrival time of Chl excitation adjacent to an activated Car.

Therefore, we divided the kinetics of the Car S_1 TA profile into 3 steps: (1) exciton arrival at the quenching site (2) rapid energy transfer to Car and (3) decay from Car S_1 to its ground state. This model can be described by a convolution of exciton migration and the Car S_1 decay kinetics:

$$Signal(t) = \int_0^t A(T) * Car(t-T)\, dT$$

where t is the pump-probe delay time. T is the time that exciton reaches any quenching site and forms Car S_1. A(T) is a population function describing the population of newly formed Car S_1 at time T. Car(t-T) is a decay function for Car S_1 relaxation, depicting the decay over time (t-T) of Car S_1 newly formed at T.

Here, we replaced $Car(t-T)$ with a single exponential decay function with a 9 ps lifetime as the relaxation of Zea S1. A(T) is described by a rise-decay function $A(T) = \sqrt{T}\, e^{-kT}$, where k is a rate parameter for optimizing convolution. Our population function is expected to overestimate the rise and decay rate of A(T). A more accurate population function can be obtained from more sophisticated simulation methods, such as exciton migration trajectory, which may suggest a slower rise rate for A(T) and give a longer convoluted decay lifetime. However, this simple model shows how we can expect a longer lifetime for the Car S_1 signal than the solution S_1 lifetime.

In Fig. S10*A*, the convoluted profile of the Car S_1 and population decay function is aligned with the PP3 profile from TA measurement, suggesting that the Car S_1 TA profile is a convoluted signal. In Fig. S10*B*, we fit both the TA and convoluted profile with a single exponential decay function, with eventually identical decay lifetimes, indicating the similarity of the kinetics. Therefore, we expected to observe a Car S_1 lifetime in thylakoid membrane longer than that in solution or in an isolated protein environment.

In this model simulation, an A(t) function with a rapid decay time constant is used to obtain a Car S_1 signal that best matches the experimental results, suggesting that the TA measurement is mostly sensitive to the excitons relatively close to the quenching site(s) undergoing $Q_y \rightarrow S_1$ energy transfer and less sensitive to slow arriving excitations. In Fig. S10*C,* we reconstructed the PP3 TA profiles in dark and light conditions using a Chl decay profile with a 300 ps lifetime and a summation of the Car S_1 convolution profile and the Chl profile, respectively. The dark TA profile is scaled using the same procedure as the TA experiment and normalized by the signal at 50 ps, which demonstrates a similar pattern as Fig. 4*A*,*C* with an additional fast decay in the light TA profile before 50 ps and matched tail kinetics after the scaling time.

Table S1. *Arabidopsis* thaliana NPQ genes and their respective *Nicotiana benthamiana* orthologs.

***A. thaliana (At)* Gene**	**At Gene Locus**	***N. benthamiana* orthologs**	**Nb Gene Locus**
NPQ4 / PsbS	At1g44575	*PsbS1*	Niben101Scf11852g00012
		PsbS2	Niben101Scf05304g05008
NPQ1 / VDE	At1g08550	*VDE1*	Niben101Scf07893g00003
		VDE2	Niben101Scf00177g07008
LUT2	At5g57030	*LUT2-1*	Niben101Scf18343g00013
		LUT2-2	Niben101Ctg13249Ctg00004*
			Niben101Ctg15093Ctg00004*

* Indicates a manually assembled gene model that was split across two draft contigs.

Table S2. gRNA spacer sequences and relative target sites in *N. benthamiana* NPQ genes.

Gene	Position from ATG	Location	Spacer Sequences (5' -> 3')
PsbS1	+331 : +351<br>+422 : +442	Exon 2<br>Exon 2	ACAGGTTGTACCAAAGCCAA
PsbS2	+351 : +371<br>+422 : +442	Exon 2<br>Exon 2	GTTGGCCGTGTTGCTATGAT
VDE1	+1983 : +2003<br>+2088 : +2108	Exon 4<br>Exon 4	GGGAAATGGTTCATAACTCG
VDE2	+2503 : +2523<br>+2608 : +2628	Exon 5<br>Exon 5	TGGAGAATACGGACACCTGA
LUT2-1	+2150 : +2170<br>+2298 : +2318	Exon 3<br>Exon 4	TAGTCGCCATTTACTGCACG
LUT2-2	+240 : +260<br>+389 : +409	Exon 2<br>Exon 3	ATCTTAACTCGAAAGTGGAT

Table S3. Homozygous, Cas9-free knockout alleles isolated in *N. benthamiana*.

T_0 Parent	Cas9-free T_1 Progeny	*PSBS1*	*PSBS2*	Mutant ID in this study
PsbS_ko-1	3	-8bp	-1bp	*psbs1 psbs2 (npq4)*
PsbS_ko-1	63	-8bp		*psbs1*
PsbS_ko-3	20, 39, 57	-7bp	-1bp	
PsbS_ko-3	59	-7bp	-2bp	
PsbS_ko-4	2, 51, 86	-8bp	-4bp	
PsbS_ko-4	10	-8bp	-2bp	
PsbS_ko-4	82	-2bp	-2bp	
PsbS_ko-5	9, 38, 52, 54	-2bp	-1bp/-1bp	
**T_0 Parent**	**Cas9-freeT_1 Progeny**	***VDE1***	***VDE2***	**Mutant ID in this study**
VDE_ko-2	3, 14, 37, 39, 42	-11bp	-1bp	*vde1 vde2 (npq1)*
VDE_ko-2	23, 24	+1bp	+1bp	
VDE_ko-3	4, 37, 181	+1bp		*vde1*
VDE_ko-3	123, 124, 146		-1bp	*vde2*
VDE_ko-7	19, 25, 36, 43	-1bp	+1bp	
**T_0 Parent**	**Cas9-freeT_1 Progeny**	***LUT2-1***	***LUT2-2***	**Mutant ID in this study**
LUT2_ko-6	51, 65, 69	-1bp	+1bp/+1bp	*lut2-1 lut2-2 (lut2)*
LUT2_ko-7	20	+1bp	+1bp	

Table S4. Primers for genomic DNA amplification of *N. benthamiana* NPQ genes and Cas9.

**Oligo ID**	**Oligo (5' -> 3')**	**Fragment Size**	**Tm**
oNb1 PsbS_1.F	GGCAGGGAGGCAAATACTAAC	308 bp	58
oNb2 PsbS_1.R	TTTCGTTTACCGCCTTTC		
oNb3 PsbS_2.F	CCAAAGCTCCTGCCAAAAAGG	428 bp	58
oNb4 PsbS_2.R	AGTTAAATGGAACGTCCGTGC		
oNb5 LUT2_1.F	CAACAAACAGAACCTCTTGTTC	500 bp	58
oNb6 LUT2_2.F	GAAACATCTTGTTCTCTGGAGC	500 bp	58
oNb7 LUT2.R	CCAGATGCAACAGTGACAAA		
oNb8 VDE.F	CTAATATGCTGGAGTGATTCTGC		
oNb9 VDE_1.R	GCACACAGAGATACGGAAC	458 bp	58
oNb10 VDE_2.R	CTATCAGCAAGGTTTAATCCAGC	567 bp	58
oNb11 Cas9.F	CGAAGAGGGCATCAAAGAG	410 bp	58
oNb12 Cas9.R	GCTGTCTCTTGATGAAGCC		

Table S5. The diffusion-related parameters for high-order nonlinear TA profiles of WT thylakoid membrane under dark and light conditions.

**Condition**	k/nm^3fs^{-1}	$D\ /10^{-2}cm^2s^{-1}$	$L_D\ /nm$	$\frac{L_{D,Light}}{L_{D,Dark}}$
**Dark**	0.042 ± 0.007	1.7 ± 0.3	62 ± 6	**69.1%**
**Light**	0.026 ± 0.004	1.0 ± 0.1	43 ± 3	

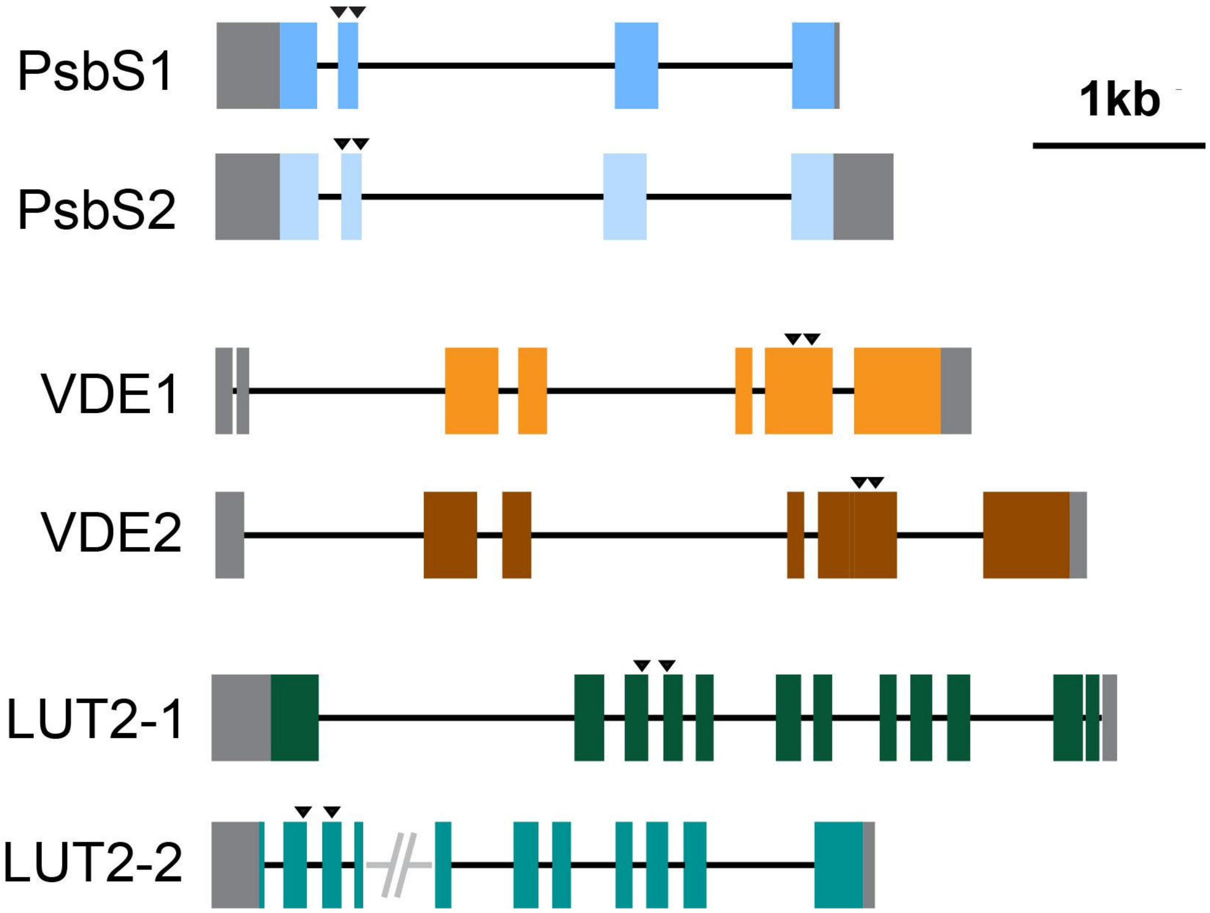

Figure S1. *Gene models and target sites for N. benthamiana mutagenesis.*

Gene models were assembled from the SolGenomics Nb-1 draft genome[15], numbered within each pair by highest identity to their respective Arabidopsis ortholog. Exons are shown by colored boxes, introns by black lines, and untranslated regions in gray. gRNA spacers are marked by black triangles. Gray slashes in *LUT2-2* indicate a gap in the draft genome contigs.

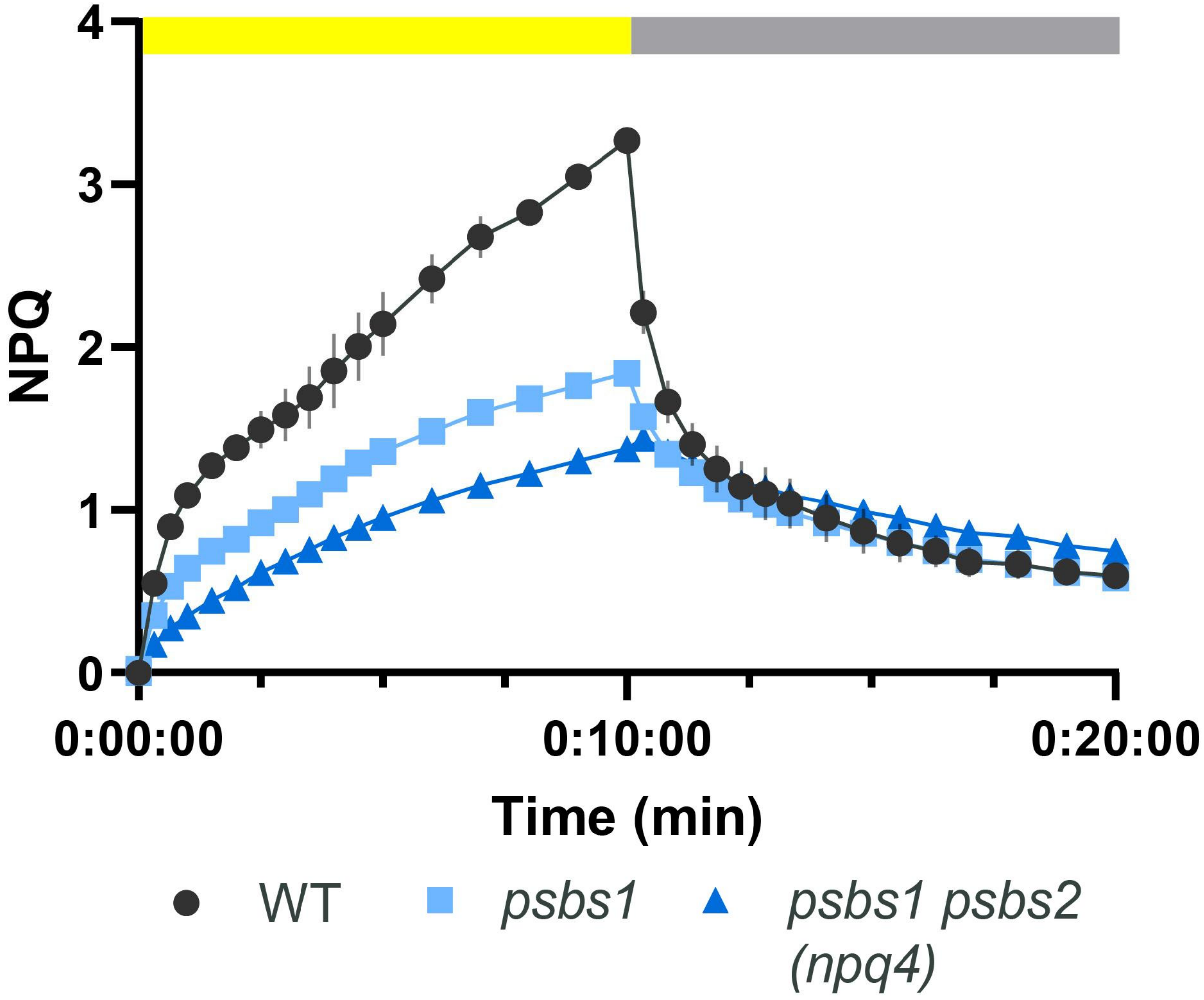

Figure S2. *NPQ kinetics of WT, psbs1, and psbs1 psbs2 (npq4).*

NPQ was measured using an Imaging-PAM Maxi (Walz) pulse-amplitude modulation fluorometer during a sequence of 10 min high light (1000 µmol photons m^{-2} s^{-1} of blue light) and 10 min of darkness (0 µmol photons m^{-2} s^{-1}). Data from WT (n=3, black circles), *psbs1* (n=6, light blue squares), and *psbs1psbs2/npq4* (n=8, dark blue triangles) are shown as means ± 1 SEM.

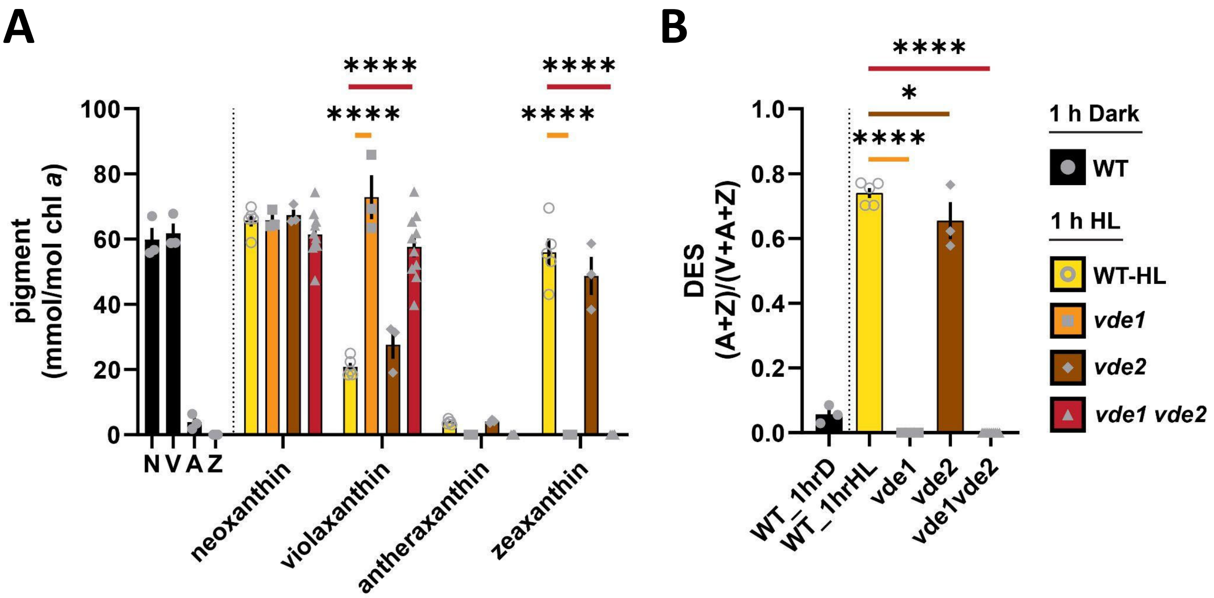

Figure S3. *Changes in xanthophyll pigment profile of WT, vde1, vde2, and vde1 vde2* mutants *after 1 h at 1500 µmol photons m^{-2} s^{-1}.*

(***A***) Concentrations of neoxanthin (N), violaxanthin (V), antheraxanthin (A), and zeaxanthin (Z) normalized to chlorophyll *a*. Data for dark-acclimated WT (n=3, black, circles) are included as a baseline for genotypes acclimated to 1 h high-light: WT-HL (n=5, yellow bars, open circles), *vde1* (n=3, orange bars, squares), *vde2* (n=3, brown bars, diamonds), and *vde1vde2* (n=10, red bars, triangles). **(*B*)** De-epoxidation state (DES) calculated as (Ant + Zea)/(Vio + Ant + Zea). Data shown as means ± 1 SEM. Pairwise significance against WT-HL was determined by ordinary two-way ANOVA (α=0.05) using Dunnett's test for multiple comparisons against with significance denoted by asterisks (*p≤0.05, ****p<0.0001).

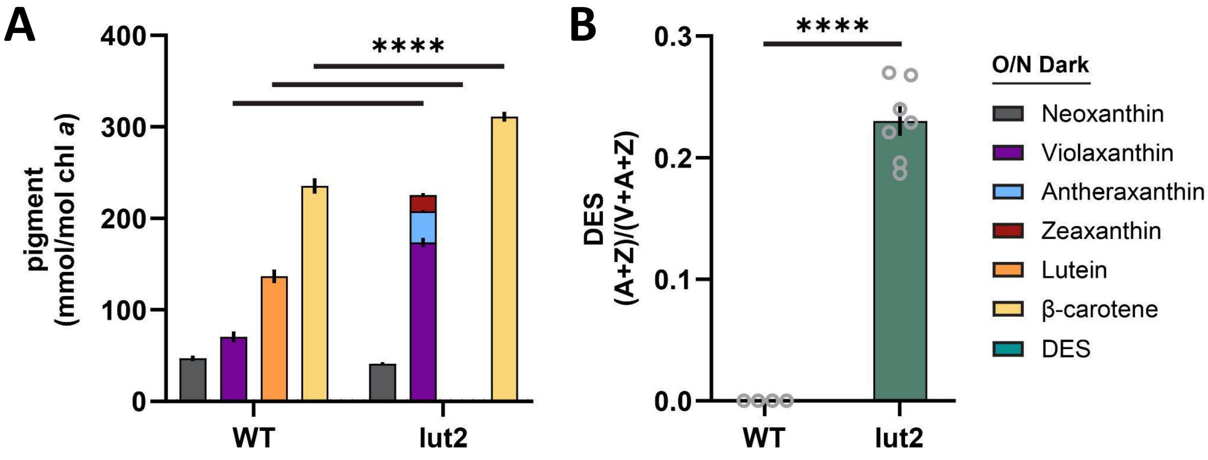

Figure S4. *Pigment profiles of WT and lut2-1 lut2-2 (lut2) after overnight dark acclimation.*

(***A***) Chlorophyll *a* normalized carotenoid abundances of WT and *lut2* leaves following overnight dark acclimation: neoxanthin (gray), violaxanthin (purple), antheraxanthin (blue), zeaxanthin (red), lutein (orange) and β-carotene (yellow). (***B***) De-epoxidation state (DES, teal) calculated as (Ant + Zea)/(Vio + Ant + Zea). Data for WT (n=4) and *lut2* (n=7) are shown as means ± 1 SEM. Pairwise significance against WT was determined by ordinary two-way ANOVA (α=0.05) using Dunnett's test for multiple comparisons against with significance denoted by asterisks (****p<0.0001).

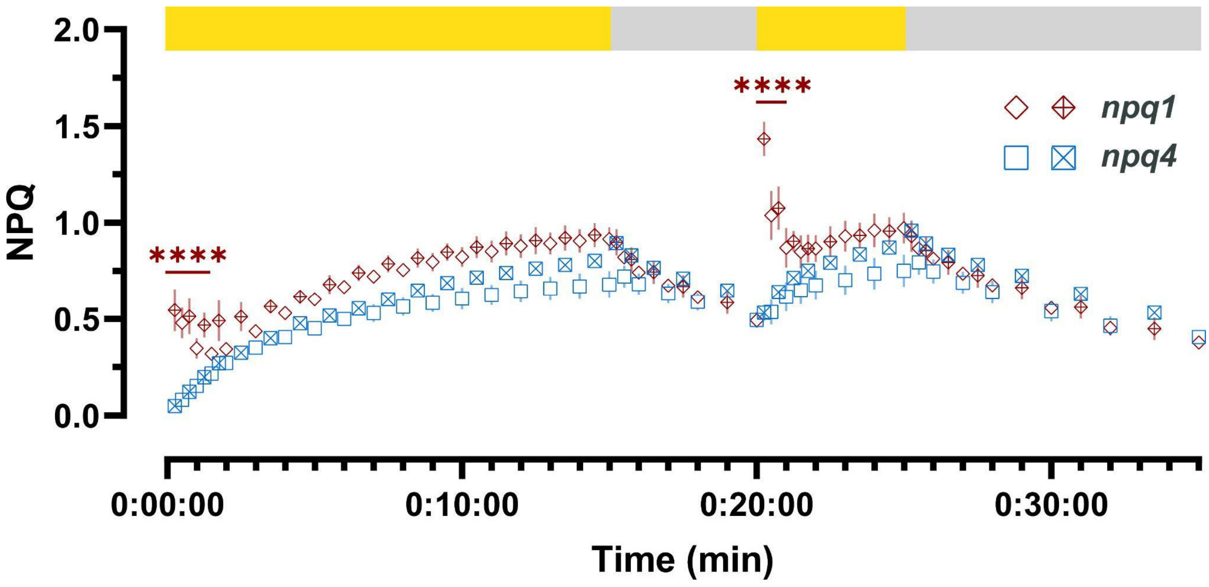

Figure S5. *Differences in NPQ induction between npq1 and npq4 mutants at 750 µmol photons m^{-2} s^{-1}.*

NPQ kinetics of *npq1* and *npq4* at 15 s resolution using two staggered measurement frequencies (n=3 each, n=6 per genotype). Pairwise significance was determined by ordinary two-way ANOVA (α=0.05) using Dunnett's test for multiple comparisons against WT with significance denoted (****p<0.0001).

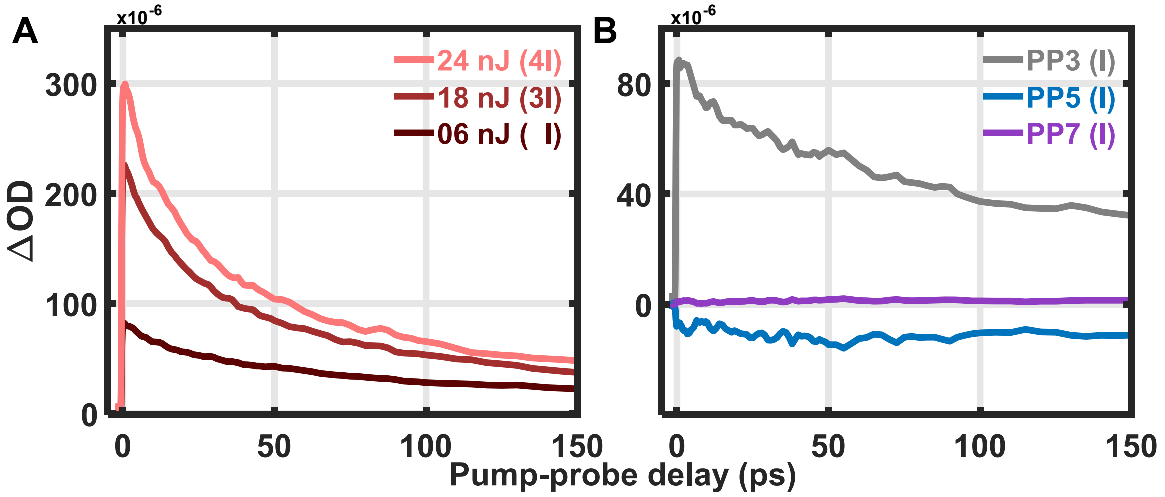

Figure S6. *Isolation of high-order nonlinear signals from WT thylakoid TA profiles using pump-intensity cycling method*.

(***A***) Temporal evolution of pump-probe signal in dark-acclimated WT thylakoid membranes probed at 540 nm and pumped at 675 nm with three different pulse energies: 6 (*I*), 18 (3*I*), and 24 (4*I*) nJ. (***B***) Isolated third (PP3), fifth (PP5) and seventh (PP7) order nonlinear signals corresponding to a 6 nJ (*I*) pump pulse energy evaluated using equation 1.

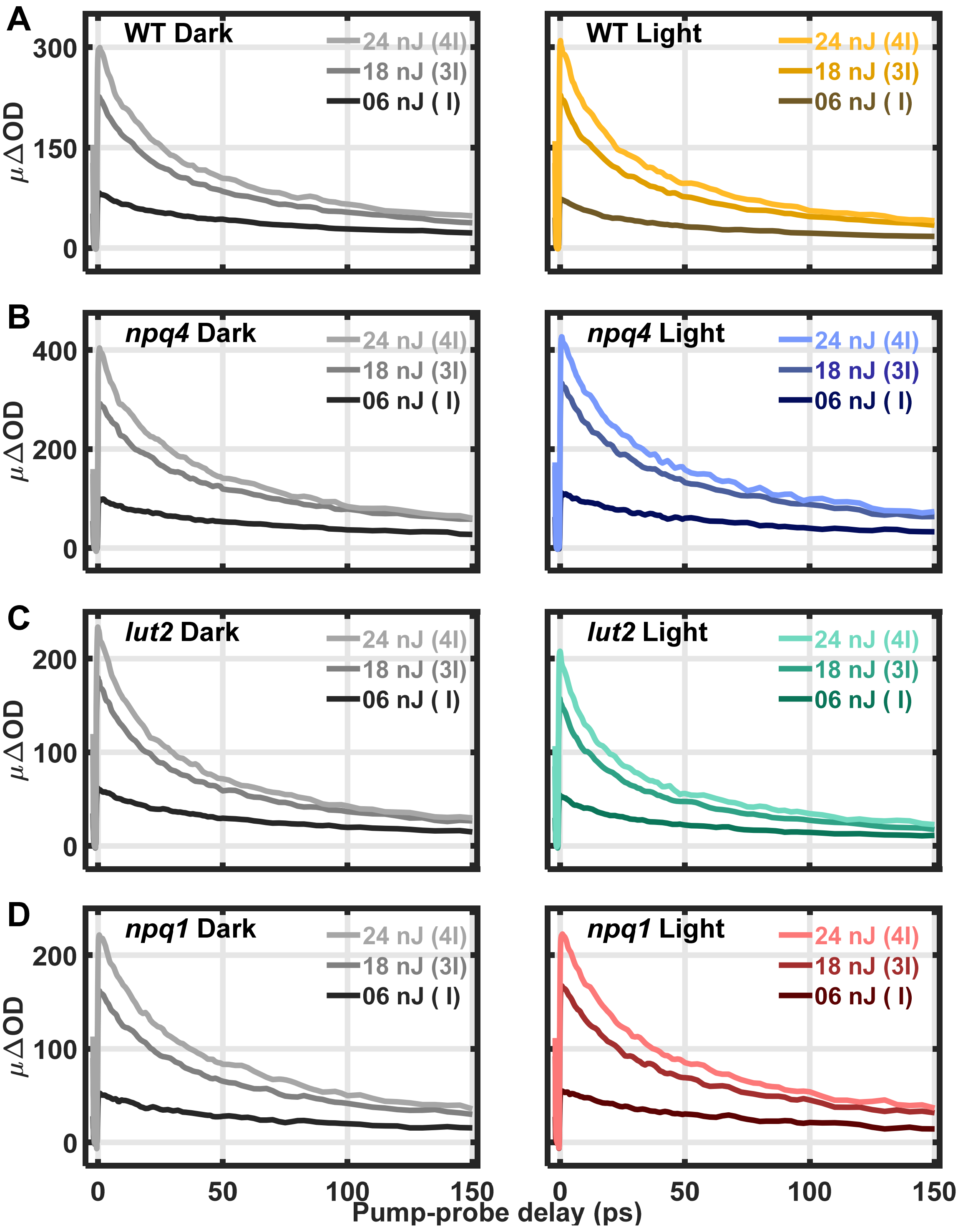

Figure S7. *Complete sets of intensity-cycling-based measurements.*

TA kinetic profiles for (***A***) WT and (***B***) *npq4* (***C***) *lut2* and (***D***) *npq1* thylakoid membranes probed at 540 nm and pumped at 6,18, and 24 nJ under (**left**) dark and (**right**) high light conditions.

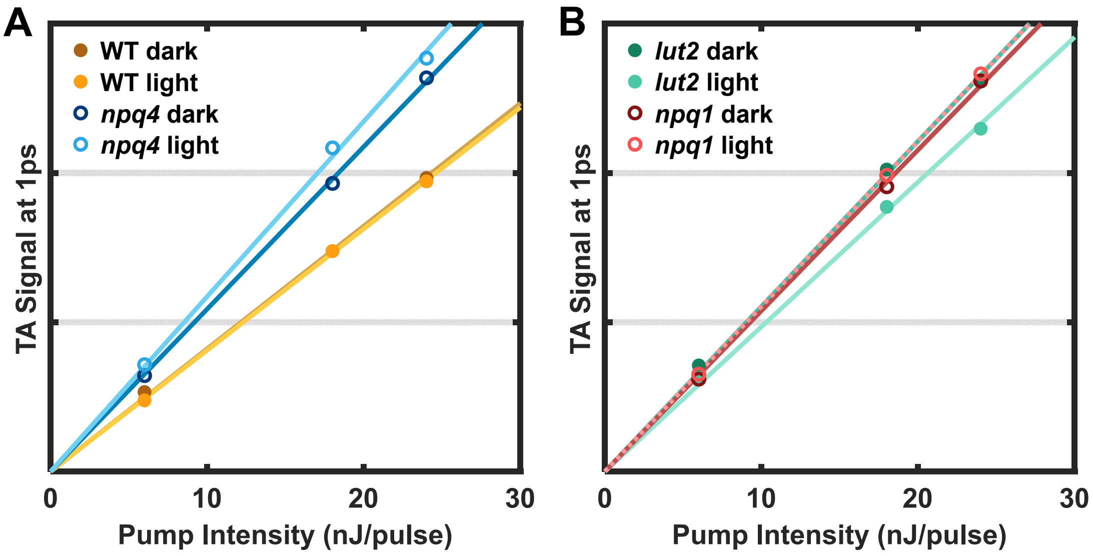

Figure S8 *Pump intensity dependence of WT and mutant thylakoid TA signal*.

Pump intensity dependence of 540 nm TA signal at 1 ps for (***A***) WT and *npq4*, (***B***) *lut2* and *npq1* thylakoid membranes under dark and high light conditions.

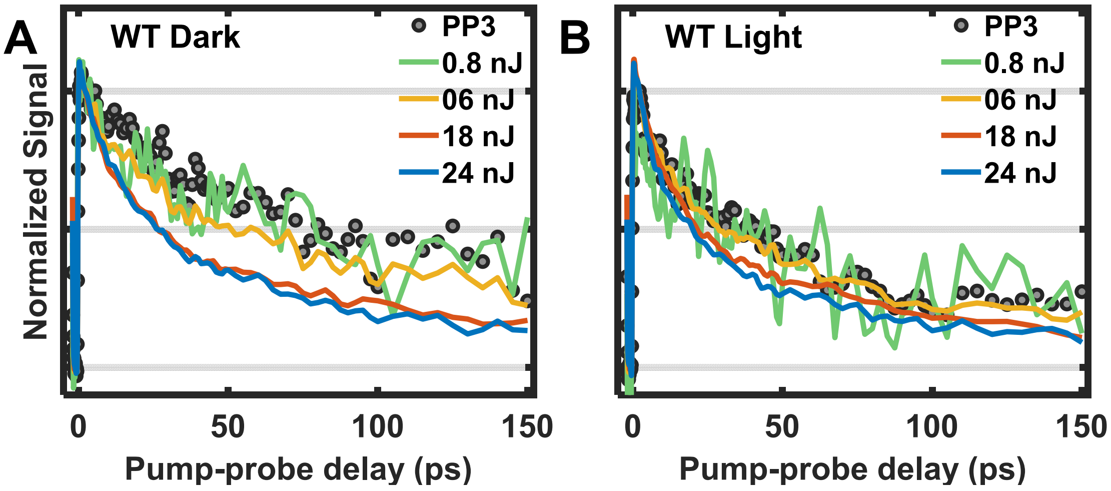

Figure S9. *Normalized PP3 kinetic profile at 6 nJ and TA profiles with various pump intensity.* Comparison between extracted PP3 and the TA signal pumped at 0.8 to 24 nJ under (***A***) dark and (***B***) light conditions.

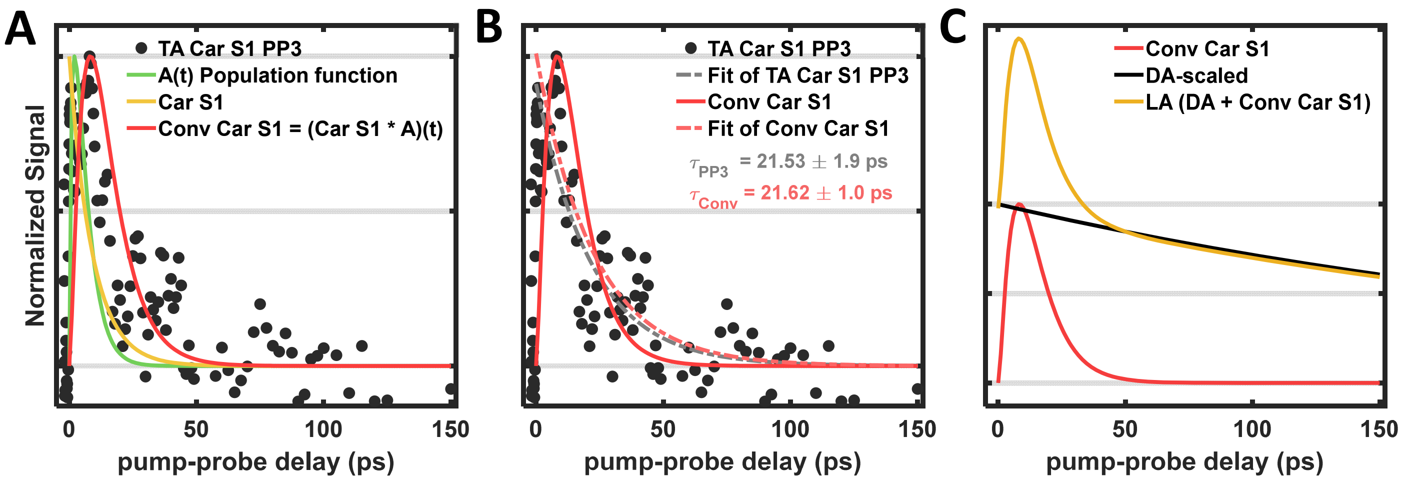

Figure S10. *Comparison between Car S_1-S_n PP3 TA profile (black dot) and simulated convolution signal (red) for WT thylakoids*.

(***A***) The Car S_1 convolution (red) of Car S_1 decay profile (yellow) and the population function A(t) of newly populated Car S_1 with an optimized decay rate 1/4.1 ps^{-1} (green). (***B***) Single exponential decay fitting of the TA Car S_1 PP3 profile (gray dash) and simulated convolution profile (red dash). Each profile except the fitting results is normalized by its maximum amplitude. (***C***) The simulated TA PP3 profiles in dark (black) and light (yellow) conditions. The dark profile decays with a 300 ps lifetime and is normalized to the light TA signal at 50 ps pump-probe delay. The light profile is a summation of the unscaled dark profile and the Car S_1 convolution profile.

SI References

1. Polívka, T. &Sundström, V. Ultrafast dynamics of carotenoid excited states-from solution to natural and artificial systems. *Chem. Rev.* **104**, 2021–2071 (2004).
